# Automated Steerable Path Planning for Deep Brain Stimulation Safeguarding Fiber Tracts and Deep Gray Matter Nuclei

**DOI:** 10.3389/frobt.2019.00070

**Published:** 2019-08-06

**Authors:** Alice Segato, Valentina Pieri, Alberto Favaro, Marco Riva, Andrea Falini, Elena De Momi, Antonella Castellano

**Affiliations:** ^1^Department of Electronics, Information and Bioengineering, Politecnico di Milano, Milan, Italy; ^2^Neuroradiology Unit and CERMAC, IRCCS Ospedale San Raffaele, Vita-Salute San Raffaele University, Milan, Italy; ^3^Department of Medical Biotechnology and Translational Medicine, Università degli Studi di Milano, Milan, Italy; ^4^Unit of Oncological Neurosurgery, Humanitas Research Hospital, Rozzano, Italy

**Keywords:** deep brain stimulation, path planning, steerable electrode, tractography, advanced diffusion MRI

## Abstract

Deep Brain Stimulation (DBS) is a neurosurgical procedure consisting in the stereotactic implantation of stimulation electrodes to specific brain targets, such as deep gray matter nuclei. Current solutions to place the electrodes rely on rectilinear stereotactic trajectories (RTs) manually defined by surgeons, based on pre-operative images. An automatic path planner that accurately targets subthalamic nuclei (STN) and safeguards critical surrounding structures is still lacking. Also, robotically-driven curvilinear trajectories (CTs) computed on the basis of state-of-the-art neuroimaging would decrease DBS invasiveness, circumventing patient-specific obstacles. This work presents a new algorithm able to estimate a pool of DBS curvilinear trajectories for reaching a given deep target in the brain, in the context of the EU's Horizon EDEN2020 project. The prospect of automatically computing trajectory plans relying on sophisticated newly engineered steerable devices represents a breakthrough in the field of microsurgical robotics. By tailoring the paths according to single-patient anatomical constraints, as defined by advanced preoperative neuroimaging including diffusion MR tractography, this planner ensures a higher level of safety than the standard rectilinear approach. Ten healthy controls underwent Magnetic Resonance Imaging (MRI) on 3T scanner, including 3DT1-weighted sequences, 3Dhigh-resolution time-of-flight MR angiography (TOF-MRA) and high angular resolution diffusion MR sequences. A probabilistic q-ball residual-bootstrap MR tractography algorithm was used to reconstruct motor fibers, while the other deep gray matter nuclei surrounding STN and vessels were segmented on T1 and TOF-MRA images, respectively. These structures were labeled as obstacles. The reliability of the automated planner was evaluated; CTs were compared to RTs in terms of efficacy and safety. Targeting the anterior STN, CTs performed significantly better in maximizing the minimal distance from critical structures, by finding a tuned balance between all obstacles. Moreover, CTs resulted superior in reaching the center of mass (COM) of STN, as well as in optimizing the entry angle in STN and in the skull surface.

## 1. Introduction

Deep brain stimulation consists in the stereotactic implantation of electrodes in deep brain structures to reversibly excite a functional target with high-frequency electrical impulses (Larson, 2014). This technique has been increasingly exploited to treat a variety of movement disorders, with a particular concern for the cardinal motor symptoms of Parkinson's Disease (PD) (Hickey and Stacy, 2016). DBS strategy for PD relies on stimulating the subthalamic nuclei (STNs) to keep them in constant refractoriness, thus inhibiting the indirect dopaminergic pathway.

Despite being an effective procedure, DBS trajectory planning toward STN is particularly challenging due to the critical position of the target, deeply sited and surrounded by eloquent structures. Overall, a correct positioning of DBS electrodes implies the accurate targeting of the desired deep structures and the anatomical obstacles avoidance, in order to maximize the treatment outcome while minimizing the surgery-related risk for the patient. The current surgical procedure planning is delicate and time-consuming, since stereotactic trajectories are now calculated on the basis of manually-defined target points (TPs) and entry points (EPs) that neurosurgeons should adjust using a trial and error approach (Breit et al., 2004).

Inappropriate trajectories could be lethal or life impairing and the risk of hemorrhages and seizures should not be underestimated (Larson, 2014). In fact, besides the other gray matter deep nuclei, also white matter (WM) motor fibers of the corticospinal tract (CST) critically run close to the STN and must be preserved. Magnetic Resonance (MR) Tractography enables the *in vivo* non-invasive dissection of WM fiber bundles, thus allowing to depict the entire course of eloquent tracts in the brain, including the corticospinal one. MR Tractography is based on diffusion-weighted MR imaging (dMRI), which measures the displacement of water molecules in biological tissues, preferentially oriented along the direction of the axonal fibers in WM (Castellano et al., 2017).

Automated computer assisted planning may significantly decrease calculation time and provide quantitative information about the safety and efficacy of trajectories. Specific anatomical constraints adapted to patient's anatomy can be inferred from clinical images. Despite the evident need of improving the proficiency of these automated approaches in avoiding obstacles, only standard preoperative imaging has been integrated into the DBS planners proposed in the literature until now. Remarkably, it must be highlighted that some eloquent structures such as WM fiber tracts, that are not identifiable on standard MRI but can be reconstructed by MR tractography, have increasing clinical relevance for neurosurgical preoperative planning.

Steerable electrodes have not been taken into account, even if the research community is increasingly proposing pioneering prototypes of flexible surgical instruments. In particular, the EU's Horizon EDEN2020 project aims at providing a step change in the microsurgical robotic field by delivering an integrated technology platform for minimally invasive surgery based on a high-tech programmable bevel-tip needle, where the displacement among four interlocked sections generates an offset on its tip so that the tool can follow Curvilinear Trajectories (CTs). When inserted into tissue, bevel-tip needles that are sufficiently thin exhibit the natural tendency to curve toward the tip of the bevel, due to the asymmetric force distribution applied by the tissue onto the surface area of the beveled tip (Watts et al., 2018). This effect can be exploited to steer the needle by varying the orientation of the shaft during insertion, thus the aforementioned steerable devices carry the unique potential of being adaptable to flexible surgical accesses (Liu et al., 2016; Secoli and Rodriguez y Baena, 2016; Secoli et al., 2018). The present study focuses on an electrode for DBS potentially engineered with a design mimicking the EDEN2020 programmable bevel tip needle. Accordingly, the aim of this work is to develop a planning algorithm for DBS which includes state-of-the-art MR imaging and that is able to estimate a pool of CTs for accurate targeting of the STN and concomitant avoidance of the other relevant gray matter nuclei and WM fiber tracts, ensuring a higher level of safety with respect to the standard rectilinear approach, based on Favaro et al. (2018a,b). The planner performances have been evaluated considering the minimum distance from critical gray and white matter obstacles, the efficacy of the target achievement and the minimum entry angle of the electrode with respect to the main axis of STN and with respect to the skull, in order to verify the potential advantage of the curvilinear trajectories over the rectilinear ones.

## 2. Related Work

Image-guided keyhole neurosurgery procedures require the precise targeting inside the brain, based on pre-operative CT/MRI images. A misplacement of the surgical tool from the planned trajectory may result in non-diagnostic tissue samples, uneffective treatment and/or severe neurological complications (Mascott, 2006; Shamir et al., 2011b). Consequently, it is desired to select a trajectory that is at a safe distance from critical structures such as blood vessels or motor and functional areas (Shamir et al., 2011a). Spatial visualization and segmentation of critical brain structures has been proposed as a means for enhancing the neurosurgeon's spatial perception and improving the awareness of structures surrounding the trajectory (Lee et al., 2002; Navkar et al., 2010; Bériault et al., 2012; Bick et al., 2012).

Blood vessel analysis plays a fundamental role in neurosurgery (De Momi et al., 2013; Faria et al., 2014; Essert et al., 2015) both for diagnosis, treatment planning, and execution. Blood vessel segmentation is necessary for their avoidance in performing path planning. Automatic or semiautomatic methods can support clinicians in performing these tasks. Moccia et al. provided a complete review of methods, datasets, and evaluation metrics (Moccia et al., 2018). For motor and functional areas avoidance, Diffusion-Tensor Imaging (DTI) tractography is widely used to map structural connections of the human brain *in vivo*. Abhinav et al. presented the technological advances leading up to the development of DTI and more advanced techniques aimed at imaging the white matter (Thomas et al., 2014). Different automatic algorithms have been proposed for minimally invasive neurosurgery, mainly for Stereoelectroencephalography (SEEG), Deep Brain Stimulation (DBS) and needle biopsies with the main goal of assisting the surgeon during the planning phase (Scorza et al., 2017).

Automated computer assisted planning solutions for DBS, computing Rectilinear Trajectories (RTs) for currently used rigid electrodes, have been presented and intensely discussed in the literature. For instance, Essert et al. extended the approach of RT calculation to an analytical description of the risk factors and suggested an additional qualitative test (Essert et al., 2012). Liu et al. validated the automatic planning method with multiple surgeons and different targets for DBS applications (Liu et al., 2014). De Momi et al. developed a method that provides the neurosurgeons with a planning tool able to maximize the distance from vessels, to avoid the sulci as entry points and to optimize the angle of guiding screws (De Momi et al., 2014). In other two studies, the authors proposed a hybrid method working by associating information of the expected risk in the form of a color map (Shamir et al., 2010, 2012). This allowed for the intuitive selection of an entry point for the desired surgical trajectory, then proposing an automatic trajectory plan. Another group developed a system for computer-assisted preoperative selection of target points and for the intraoperative adjustment of them (D'Haese et al., 2005).

Going beyond RTs, the safeguarding of critical structures could be further implemented with new models of electrodes able to navigate along CTs, that will overcome limitations related to straight and non-malleable paths. Existing steerable needle concepts can be classified in seven different groups, as summarized in a recent work (van de Berg et al., 2015): bevel tip, base manipulation, optically controlled needle, pre-curved stylet, active cannula, tendon actuated tip, and programmable bevel tip.

Regarding CT approaches, some solutions can be found in keyhole neurosurgical scenario. Duindam et al. proposed a 3D motion planning for a steerable needle as a dynamical optimization problem with a discretization of the control space using inverse kinematics (Duindam et al., 2018). Other solutions proposed in literature can be divided in two main categories: graph-based and sampling-based methods. Two examples of graph search methods are Dijkstra's algorithm, which aims at finding the shortest path between a node and all other nodes in the graph (Dijkstra, 1959) and A*, that is an improved version of the Dijkstra's method, using an heuristic function (Hart et al., 1968). Park et al. presented a diffusion-based motion planning for a non-holonomic flexible needle based on a probability map (Park et al., 2005). Although graph-based methods are relatively simple to implement, they require a considerable computational time as the environment becomes more complex (Bellman, 1966).

Sampling-based solutions are the current trend for generic single-query path planning problems. Remarkably, Rapidly-exploring Random Tree (RRT) (LaValle and Kuffner, 2000) is an exploration algorithm for quickly searching high-dimensional spaces and it's much more efficient than brute-force exploration of the state space. Several authors (Rodriguez et al., 2006; Knepper and Mason, 2009) proposed different exploration algorithm for RRTs with randomly sampled C space and deterministic control space. Branicky et al. extended the RRT-based method for a motion planning approach considering a system with a hybrid configuration space and constraints (Branicky et al., 2003). Particularly interesting, in this regard, is the study of Favaro et al. (2018a) proposed in the context of EDEN2020, which improved the approaches described in previous works applying an informed RRT algorithm, designed to meet the catheter kinematic constraints and non-holonomicity and to guarantee a high reliable level of obstacle-avoidance capability, crucial for the intended neurosurgical application.

To our knowledge, there is no algorithm in the literature that calculates automatically curvilinear safe paths for DBS integrating tractography reconstructions. Thus, following CTs may enhance the chances to obtain an optimal targeting of the STN with the proper anatomical obstacles avoidance, since flexible electrodes can mitigate limitations of their rigid counterparts through their ability to steer along CTs (Favaro et al., 2018b).

## 3. Materials and Methods

### 3.1. Surgeon's Input and Data Processing

As first step, the surgeon is asked to select the desired entry point (EP) on the brain cortex, the target structure (TS) within the brain, corresponding to the STN and, optionally the target point (TP). This latter, if not specified, will coincide with the center of mass of the STN. The anatomical obstacles (AOs) are segmented and a distance map is computed (Danielsson, 1980).

The system delineates an entry area EA around the EP, excluding the sulci as possible entry area because of the presence of cortical blood vessels, thus preventing possible hemorrhages (De Momi et al., 2014). A mesh decimation is performed over the EA and a pool of 10 feasible entry points EP_*i*_, *i* ∈ 1, .., 10 is defined.

### 3.2. Path Planning ∀ EP_*i*_

**Figure 1:**
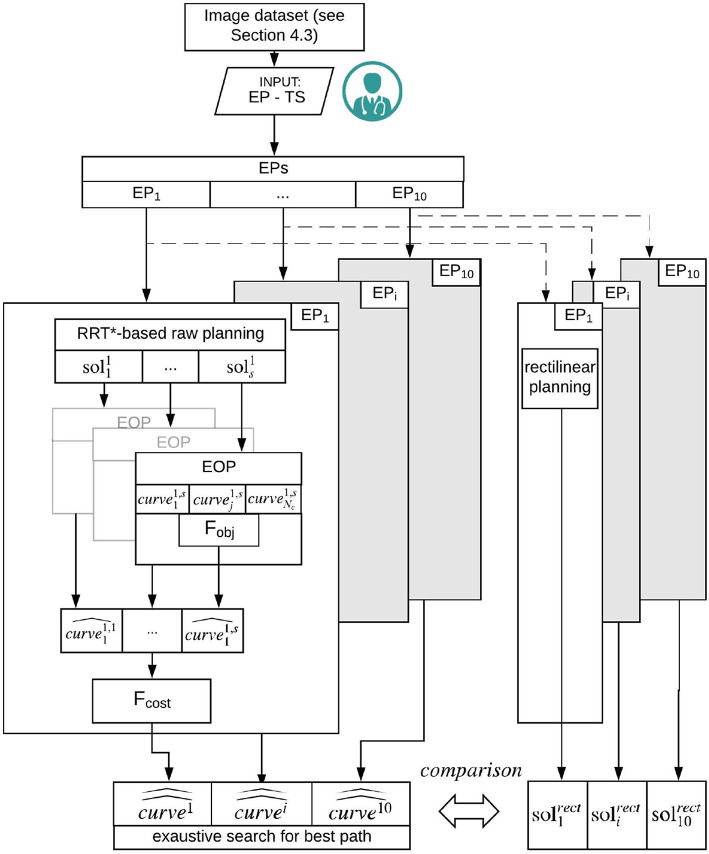
Schematic representation of the workflow. From the segmented image dataset, 10 EPs are selected. On each EP, the RRT based raw planning is applied computing *s* {$sol_{s}^{i}$} solutions (section 3.2.1). Subsequently, an EOP is computed based on *Fobj* to be minimized. A number of feasible solutions {$\hat{curve^{i,s}}$} is generated (section 3.2.2). Finally for each EP_*i*_ the best path {$\hat{\hat{curve^{i}}}$} is computed by running a cost function *Fcost*, to be minimized, over the set of solutions generated by the Exaustive search for best path (section 3.2.3).

Our path planner method consists in three main steps: Path planning, described in section 3.2.1, where a set of piece-wise linear feasible paths is computed from each EP_*i*_ to the target point, Path approximation and optimization, described in section 3.2.2, where an evolutionary optimization procedure generates smooth paths, reduces their lengths and optimizes the insertion angle with respect to the target main axis and Exhaustive search for the best path, reported in section 3.2.3, where an exhaustive search is performed over the set of paths for determining the best planning solution. The entire workflow is described in Figure 1.

#### 3.2.1. RRT*-Based Raw Planning

At first, an ellipsoidal volume H is built, having the EP and TP as foci. The focal length of the ellipsoid is the Euclidean distance between the EP and the TP and corresponding to the minimum possible path length, the minor axis of the ellipsoid is set to a predefined value equal to 10 *mm*. In this way, the original search workspace, consisting in the entire patient's brain, is bordered within a confined region, H.

A batch of uniformly-sampled 3D points in H is gradually provided to an RRT*-based planning algorithm (Gammell et al., 2015), to build a connected graph of vertices and obstacle-free edges.

As a first path able to connect the EP_*i*_ to the TP is detected, the solution is stored. Subsequently, the RRT* keeps adding new points in H. As a new, shorter solution is discovered, the graph is pruned and the major axis of H is reduced to the length of the new solution resulting in focusing the search within a smaller space. This new piece-wise linear pathway is stored as well. A number of paths $sol_{s}^{i}$, $s\in 1,\ldots ,N_{s}^{max}$ is thus defined as a sequence of vertices **P**_*k*_ (*k* = 1…*N*_*v*_), where *N*_*v*_ is the number of vertices, such that:

(1) $$\begin{array}{l} sol_{s}^{i}=\left\{{\text{P}}_{k}^{i,s}\in {\mathbb{R}}^{3}\right\} \end{array}$$

where $N_{s}^{max}$ is a predefined upper limit of possible solutions discovered for the specific EP_*i*_ with *i* ∈ 1, .., 10, ${\text{P}}_{1}^{i,s}=$ EP_*i*_ and ${\text{P}}_{N_{v_{i}}}^{i,s}=$ TP. The reader is referred to Favaro et al. (2018a), Favaro et al., (under submissiion) for further details.

#### 3.2.2. Evolutionary Optimization Procedure (EOP)

An Evolutionary Optimization Procedure (EOP) is run (Favaro et al., under submission). The vertexes of each piece-wise linear solution $sol_{s}^{i}$ are used to define a population $\left\{curve_{j}^{i,s},j=0,\ldots ,N_{c}\right\}$ of Non-Uniform Rational Beta Splines (NURBS) by assigning different random weights to each vertex. $\left\{curve_{j}^{i,s}\right\}$ is made to evolve according to the objective function to minimize (*F*_*obj*_). This results in pulling (pushing) a $curve_{j}^{i,s}$ closer to (far from) the vertexes so that to optimize the curve in accordance with *F*_*obj*_. Hyper-parameters used in the EOP are reported in Table 1.

**Table 1:** Parameters used for the EOP.

**EOP parameters**			
**N_***c***_**	**N_***i***_**	**p_***cross***_**	**p_***mut***_**
20	50	0.5	0.1

*With the exception of N_c_, which has been set empirically and represents the number of NURBS individuals composing the population of each piece-wise linear solution $sol_{s}^{i}$, the number of EOP iterations N_i_, the cross-over probability p_cross_ and the mutation probability p_mut_ are taken from Jalel et al. (2015)*.

The best trajectory obtained will:

1. Minimize the number of points of the path intersecting an obstacle.
2. Minimize the maximum curvature of the path *k*_*path*_, to respect kinematic constraints such as the maximum curvature of the electrode (*K*_*max*_).
3. Minimize the length of the electrode *l*.
4. Minimize the standard deviation of curvature values, in order to obtain a smoother path.
5. Optimize the orientation of the electrode depending on TS shape.

Thus *F*_*obj*_ is defined as:

(2) $$\begin{array}{l} F_{obj}\left(\left\{curve_{j}^{i,s}\right\}\right)=\beta_{1}\cdot \;\#p_{unsafe}\;+\;\beta_{2}\cdot \#p_{unfea}\;+\;\beta_{3}\cdot l\;+\;\beta_{4}\cdot \;SD\\ \;+\beta_{5}\cdot \;\alpha \end{array}$$

where:

- *#p*_*unsafe*_ is the number of points **P**_*unsafe*_ ∈ {$curve_{j}^{i,s}$} whose 3D coordinates are internal to the point cloud describing each AOs, such that:
  (3) $$\begin{array}{l} \left\{{\text{P}}_{unsafe}\right\}:\left\{curve_{j}^{i,s}\cap cloud_{AOs}\right\} \end{array}$$
  where {*cloud*_*AOs*_} is the set of 3D points representing the obstacle space with the AO surface.
- *#p*_*unfea*_ is the number of points **P**_*unfea*_ ∈ $\left\{curve_{j}^{i,s}\right\}$ whose curvature, $k_{path}=curve_{j}^{\prime \prime i,s}$ (calculated as the second derivative of $curve_{j}^{i,s}$; Favaro et al., under submission), exceeds the maximum curvature achievable by the needle (*K*_*max*_), such that:
  (4) $$\begin{array}{l} \left\{{\text{P}}_{unfea}\right\}:\left\{k_{path}>K_{max}\right\} \end{array}$$
- *l* is the total path length of {$curve_{j}^{i,s}$}, such that:
  (5) $$\begin{array}{l} l\left(\left\{curve_{j}^{i,s}\right\}\right)=\int_{EP_{i}}^{TP}∥\left\{curve_{j}^{i,s}\left(u\right)\right\}∥du \end{array}$$
  where *u* ∈ [0, 1] is the independent variable used to define the NURBS curve in parametric form, The reader is referred to Favaro et al., (under submission) for further details.
- SD is the standard deviation of the curvature, *k*_*path*_, such that:
  (6) $$SD=\sqrt{\frac{1}{N_{s}}\sum_{i=1}^{N_{s}}{\left(k_{path}-\bar{k_{path}}\right)}^{2}}$$
  where *N*_*s*_ represents the number of samples of $\left\{curve_{j}^{i,s}\right\}$ that depend upon the discretization of *u* ∈ [0, 1].
- As the STNs have an anisotropic shape, for each STN the longitudinal axis is defined computing Principal Component Analysis (PCA) of the STN point cloud segmentation and used as desired trajectory for the distal part of the needle. Specifically, the entry angle α between the distal part of the needle (the one inserted in the STN) and the longitudinal axis of the STN is computed as:
  (7) $$\begin{array}{l} \alpha =\text{arccos}\left({\hat{l}}_{dist}\cdot {\hat{l}}_{STN}\right) \end{array}$$
  where ${\hat{l}}_{dist}$ is the 3D unit vector representing the entry direction of the distal part of the needle. ${\hat{l}}_{STN}$ represents the 3D unit vector of the 1st PCA component.
- The values of the weight are empirically defined and reported in Table 2.

**Table 2:** Parameters used in the experimental setup.

**PBN parameters**		**Objective function**					**Cost function**							**Check function**
**⌀[mm]**	***K*_*max*_ [*mm*^−1^]**	**β_1_**	**β_2_**	**β_3_**	**β_4_**	**β_5_**	**κ_1_**	**κ_2_**	**κ_3_**	**κ_4_**	**κ_5_**	**κ_6_**	**κ_7_**	**$\theta_{max}\left[{}^{\circ}\right]$**
1.3–2.5	0.015-0.055	5	5	0.1	1	0	0	0	0	0	1	0.5	0.2	0
1.3	0.015	5	5	0.1	1	2	0.5	0.1	0.1	0.1	1	0.5	0.2	30

*Line1 corresponds to feasibility study, while line2 to validation of RTs vs CTs. From the left to the right, the PBN diameter (⌀) and maximum degree of curvature (K_max_) are reported, followed by the values of the weight used in the Objective and the Cost functions. The value assigned to the thr_max_ used in the Check functions is shown. Lastly, the values of the threshold density is also reported*.

Minimizing $F_{obj}\left(\left\{curve_{j}^{i,s}\right\}\right)$, through a preset number of iterations, allows each new offspring of the EOP, {$curve_{j}^{i,s}$}, to move toward the path optimality. {$\hat{curve_{1}^{i,s}}$} represents the best $C^{2}$, obstacle-free path able to connect EP_*i*_ to the TP starting from the piece-wise linear solution *s*.

#### 3.2.3. Exhaustive Search for Best Path

Among the optimized solutions {$\hat{curve^{i,s}}$} that defined the feasible optimized trajectories from EP_*i*_ to TP, the best one is identified through a cost function to minimize, *F*_*cost*_, expressed as follows:

(8) $$F_{cost}(\{\hat{curve^{i,s}}\})=\left\{ \begin{array}{l} \begin{array}{ll} \infty & \text{ if }d_{min}\leq 0\\ \infty & \text{ if }k_{path}>K_{max} \end{array}\\ \kappa_{1}\frac{1}{d_{THA}}+\kappa_{2}\frac{1}{d_{GP}}+\kappa_{3}\frac{1}{d_{CN}}+\kappa_{4}\frac{1}{d_{CST}}\\ +\kappa_{5}\frac{1}{d_{min}}+\kappa_{6}\frac{1}{\bar{d}}+\kappa_{7}\frac{k_{path}}{K_{max}}\text{otherwise} \end{array}\right.$$

where, given the euclidean distance *d*_*e, o*_, defined as:

(9) $$\begin{array}{l} d_{e,o}=∥P_{e}-P_{o}∥ \end{array}$$

with *P*_*e*_ = {*P*}_*path*_, with *e* ∈ 1, …, *N*, is the set of points of the calculated *curve*^*i, s*^ and *P*_*o*_ = {*P*}_*AO*_, with *o* ∈ 1, …, *M*, is the set of 3D points representing the obstacle *AO*, with *AO* = {*THA, GP, CN, CST*}.

- *d*_*min*_ is the minimum distance calculated over the whole length, (*l*), of the {$\hat{curve^{i,s}}$} with respect to all the AOs, such that:
  (10) $$\begin{array}{l} d_{min}=\text{min}\left\{d_{e,o}\right\}\forall P_{e},\forall P_{o} \end{array}$$
- $\bar{d}$ is the average distance calculated over the whole length, (*l*), of the {$\hat{curve^{i,s}}$} with respect to all the AOs, such that
  (11) $$\begin{array}{l} \bar{d}=\frac{1}{N\cdot M}\overset{N}{\underset{e=1}{\sum }}\overset{M}{\underset{o=1}{\sum }}d_{e,o} \end{array}$$
- *d*_*THA*_, *d*_*GP*_, *d*_*CN*_, *d*_*CST*_ represent the sum of *d*_*min*_ and $\bar{d}$ with respect to the 4 most critical AO taken singularly: thalamus (THA), globus pallidus (GP), caudate nucleus (CN), and corticospinal tracts(CST). For the sake of clarity are all defined as:
  (12) $$\begin{array}{l} d_{AO_{k}}=d_{min_{AO_{k}}}+{\bar{d}}_{AO_{k}},\forall AO_{k} \end{array}$$
  where
  $$d_{min_{AO_{k}}}=\text{min}\left\{d_{e,k}\right\},\forall P_{e},\forall P_{o,k}\in AO_{k}$$
  and
  $${\bar{d}}_{AO_{k}}=\frac{1}{N\cdot \#AO\cdot M}\overset{N}{\underset{e=1}{\sum }}\overset{\#AO}{\underset{k=1}{\sum }}\overset{M}{\underset{o=1}{\sum }}d_{e,o}$$
- Weights from κ_1_ to κ_4_ are defined by the user, according to the possibility of the surgeon to set the priorities for maintaining distances with respect to structures, while from κ_5_ to κ_7_ are empirically defined and reported in Table 2.

The output of this step is the best path from each EP_*i*_ to TP over the entire set of {$\hat{curve^{i,s}}$}, identified as:

(13) $$\begin{array}{l} \{\hat{\hat{curve^{i}}}\}=\underset{x\in \{\hat{curve^{i,s}}\}}{\text{argmin}}f(x)=\{x\in \{\hat{curve^{i,s}}\}:\\ \;f(x)=\underset{y\in {\left\{\hat{curve^{i,s}}\right\}}_{i}}{\text{min}}F_{cost}(y)\} \end{array}$$

A further surgical need is to compute a trajectory possibly aligned to the main axis of the target, especially in ellipsoidal STN, in order to cover almost all the nucleus and to increase the electrostimulation. We define θ_*max*_=30° as the maximum insertion angle with respect to skull normal acceptable for electrode placement. The insertion angle θ_*EP*_ between the proximal part of the needle (the one near the EP*i*) and the skull normal is computed as:

(14) $$\begin{array}{l} \theta_{EP}=\text{arccos}\left({\hat{l}}_{prox}\cdot {\hat{l}}_{SKULL}\right) \end{array}$$

where ${\hat{l}}_{prox}$ is the 3D unit vector representing the entry direction of the proximal part of the needle. ${\hat{l}}_{SKULL}$ represents the 3D unit vector of the skull normal. A check function *F*_*check*_(θ_*EP*_) is then computed:

(15) $$F_{check}(\theta_{EP})=\{\begin{array}{cc} discarded & \text{ if }\theta_{EP}>\theta_{max}\\ accepted & \text{otherwise} \end{array}$$

The developed system was implemented in the 3DSlicer software (4.7.0-2017-10-16) on iMac (OS-X 10.13.3 (17D47), 2,9 GHz Intel Core i5, 8GB of RAM).

## 4. Experimental Setup

### 4.1. MRI Acquisition

High-resolution MR images of ten healthy controls (mean age: 38 yo; 5M/5F) have been acquired on a 3T Ingenia CX scanner (Philips Healthcare, Best, The Netherlands). The research ethical committee of Vita-Salute San Raffaele University and IRCCS San San Raffaele Scientific Institute approved the study, and all subjects provided signed informed consent prior to MR imaging. The MRI protocol included:

- a 3D T1-weighted sagittal Fast-Field Echo with selective water excitation (Proset technique) acquired with the following parameters: repetition time/echo time [TR/TE] 12/5.9 *ms*; flip angle, 8°; acquisition matrix, 320 × 299; voxel size, 0.8 × 0.8 × 0.8 *mm*; thickness, 0.8/0 *mm* gap; SENSitivity-Encoding [SENSE] reduction factor, R = 2; 236 slices; acquisition time, 5 *min* 19 *s*;
- a simultaneous multislice Echo Planar Imaging (EPI) axial sequence for Diffusion MR Imaging (dMRI), acquired at multiple *b*-values (0, 711, and 3,000 $\frac{s}{mm^{2}}$) with diffusion gradients applied along 35 and 60 non-collinear directions and the following parameters: TR/TE 5977/78 *ms*; flip angle, 90°; acquisition matrix, 128 × 126; voxel size, 2 × 2 × 2 *mm*; thickness, 2/0 *mm* gap; SENSE factor, R = 2; Multiband factor = 2; 60 slices. Twelve b = 0 images were obtained, including one with reversed phase-encoding to estimate susceptibility-induced distortions;
- a 3D high resolution time of flight MR angiography (TOF-MRA) acquisition to visualize flow within the arterial vessels, acquired with parameters as follows: TR/TE 23/3.45 *ms*; flip angle, 18°; acquisition matrix, 500 × 399; acquired voxel size, 0.4 × 0.5 × 0.9 *mm*; reconstructed voxel size, 0.3 × 0.3 × 0.45 *mm*; thickness, 0.45/−0.45 *mm* gap; SENSE factor, R = 2; 210 slices; acquisition time, 8 *min* 33 *s*.

### 4.2. MRI Analysis and Tractography Reconstructions

From the multi *b*-value dMRI dataset, high angular resolution diffusion-weighted imaging (HARDI) volumes (60 diffusion directions, *b*-value = 3,000 $\frac{s}{mm^{2}}$) and b0 images were extracted by using the “fslsplit” and “fslmerge” tools of FMRIB Software Library (FSL, https://fsl.fmrib.ox.ac.uk/fsl/). Current distortions as well as susceptibility distortions were corrected. Diffusion tensor and fractional anisotropy (FA) maps were estimated using Diffusion imaging in Python (Dipy) software (Garyfallidis et al., 2014). MR Tractography reconstruction was based on a q-ball residual bootstrap algorithm (Berman et al., 2008), in order to fit the signal to spherical harmonics, to compute the Orientation Distribution Functions (ODFs), and to identify the primary fiber orientation.

To reconstruct bilateral corticospinal tracts (CSTs), seeding regions-of-interest (ROIs) were selected on axial images including an area of high anisotropic diffusion in the anterior part of the pons, and target regions were chosen at the level of the primary motor cortices in the precentral gyri. Maximum turning angle of 60° and FA threshold of 0.1 were used as stopping criteria for fiber tracking.

Arterial vessels have been segmented on the TOF-MRA images, in the native space of each patient, by applying an intensity threshold with 3D Slicer©.

Finally, the b0 volume from the HARDI data, the 3D T1-weighted images and the TOF-MRA images were co-registered to the MNI image volume (Ewert et al., 2017) by means of a 3D affine transformation. The transformation matrix of the b0 volume was applied to both the .trk files and NifTI binary masks of the CSTs, in order to bring the tracts in a standard reference space. Similarly, the masks of the arterial vessels were reported in the MNI space.

### 4.3. Experimental Protocol

**Figure 2:**
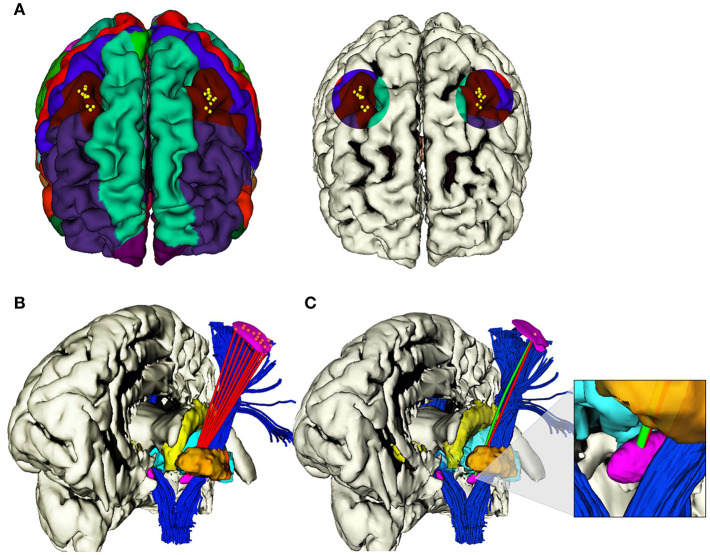
**(A)** Selected areas of 1 cm diameter in the caudal middle frontal gyrus, where *i* ∈ 1, .., 10 EP_*i*_ were determined in each hemisphere. **(B)** Example of the computation of *i* viable RTs (red) from *i* EPs to the same TP on the anterior portion of STN, left hemisphere. **(C)** Example of the computation of correspondent RT (red) and CT (green), from the same EP_*i*_ to the same *TP*_*i*_ on the anterior portion of STN, left hemisphere.

On the normalized 3D T1-weighted images previously coregistered to the MNI space, we segmented cerebral cortex, skull surface, arterial blood vessels, and ventricles by means of FreeSurfer Software, then relevant deep gray matter structures [(THA), (GP), (CN)] and the DBS target STN, by using the DISTAL atlas with 3D Slicer©. Each start and target points pair has been set as in DBS clinical practice (Figure 2; Okun, 2012).

The herein described method was tested in two different phases described in the following sections 4.3.1 and 4.3.2. All the relevant parameters used in the tests are reported in Table 2.

#### 4.3.1. Feasibility Study on Catheter Specifications

The feasibility study is aimed at computing the max diameter (EOD) and the minimum curvature (*k*) that could allow safe paths toward the TPs.

Tests were conducted bilaterally on one case-study. For each hemisphere, 2 EPs were chosen and for each point a solution path was provided. Five electrode outer diameters (EOD_*j*_) with *j* ∈ 1..5 were tested, starting from the standard 1.3 up to 2.5 *mm* range with a step of 0.3 *mm*. The value of *k* was increased stepwise from 0.015 to 0.055 *mm*^−1^ with a step of 0.010 *mm*^−1^. The performance in terms of *d*_*min*_ from the AOs was computed.

#### 4.3.2. Validation of RTs vs. CTs

The validation phase included multiple tests, performed on 10 cases, selecting 2 TPs for each hemisphere: 1 defined manually on the basis of current clinical practice and 1 in the center of mass of the STN. An EOD of 1.3 *mm* was considered and a *K*_*max*_ of 0.015 *mm*^−1^ was chosen.

From the EP_*i*_ each RT was computed by linearly connecting each EP to the related TP and solutions ${\text{sol}}_{i}^{RT}$ were obtained. Moreover, CT were obtained with the application of the method described in section 3. Finally, for every EP_*i*_, the CT solutions {$\hat{\hat{curve^{i}}}$} is compared with the standard RT ones ${sol}_{1}^{RT}$ (Figure 2).

**Figure 3:**
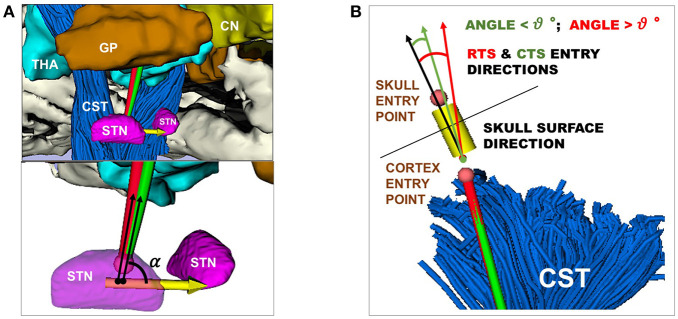
**(A)** Representation of RT (red) and CT (green) entry angle, (α), into the STN, the illustrative scene of single-case example has been taken from 3D Slicer 4.7.0 **(B)** Representation of RT(red) and CT (green) in keeping a skull entry angle < θ°, the illustrative scene of single-case example has been taken from 3D Slicer 4.7.0.

For each RT and CT solution, we calculated:

- The minimum (*d*_*min*_) and the mean ($\bar{d}$) distances with respect to all the obstacles (AOs point cloud), as described in Equations (10) and (11) (section 3.2.3). The minimum (*d*_*mi*_*n*__*AO*__) and the mean (${\bar{d}}_{AO}$) distances with respect to an obstacle taken singularly (THA, GP, CN, or CST point cloud), as described in Equation (12) (section 3.2.3).
- The STN entry angle α were computed, as described in Equation (7) and shown in Figure 3A.
- The percentage of CTs and RTs that did not exceed θ_*max*_=30°, defined as the maximum insertion angle with respect to skull normal acceptable for electrode placement (Scorza et al., 2017), as described in Equation (15) and shown in Figure 3B.

All the parameters were analyzed by means of Matlab (The MathWorks, Natick, Massachusetts, R2017b) and Graph Pad Prism 7 (GraphPad Software, La Jolla, California, USA). Lilliefors test has been initially applied for data normality. Due to the non-normality of data distribution, pairwise comparison RT and corresponding CT to any anatomical obstacles was performed with Wilcoxon matched-pairs signed rank test. Differences were considered statistically significant at *P* < 0.05. It is worth specifying that analyses have been conducted keeping data of the right hemispheres separate from the left ones, in order to respect the functional more than the anatomical variability between the two hemispheres. In fact, they are generally approached very differently in the surgical setting, depending on the patient-specific side dominance.

## 5. Results

### 5.1. Feasibility Study

**Figure 4:**
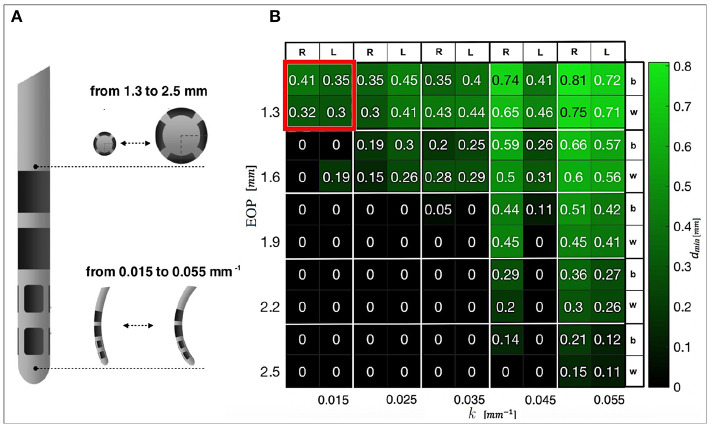
**(A)** DBS electrode prototype, in which the tip that actually releases the stimulation is shown. Examples of progressive increase in diameter and curvature are presented. **(B)** Heatmap representing the minimal distances from AOs obtained when differently designed CTs reach the anterior STN. The best (b) and worst (w) results for the left (L) and right (R) hemispheres are displayed. In red, the constrains that we selected for successive tests are highlighted.

A heatmap was generated to show the minimum curvature required by any tested diameter in order to compute safe trajectories for flexible electrodes. Figure 4A shows electrodes with different diameters [*mm*] and different maximal curvatures [*mm*] that have been tested. Figure 4B shows *d*_*min*_ with respect to AOs. A catheter with EOP of 2.3 [*mm*] allows clearance from obstacles if the curvature *k* is 0.015 [*mm*^−1^]. A curvature of 0.055 [*mm*^−1^] allows free trajectory up to 2.5 [*mm*] of diameter.

### 5.2. Validation of RTs vs. CTs

**Figure 5:**
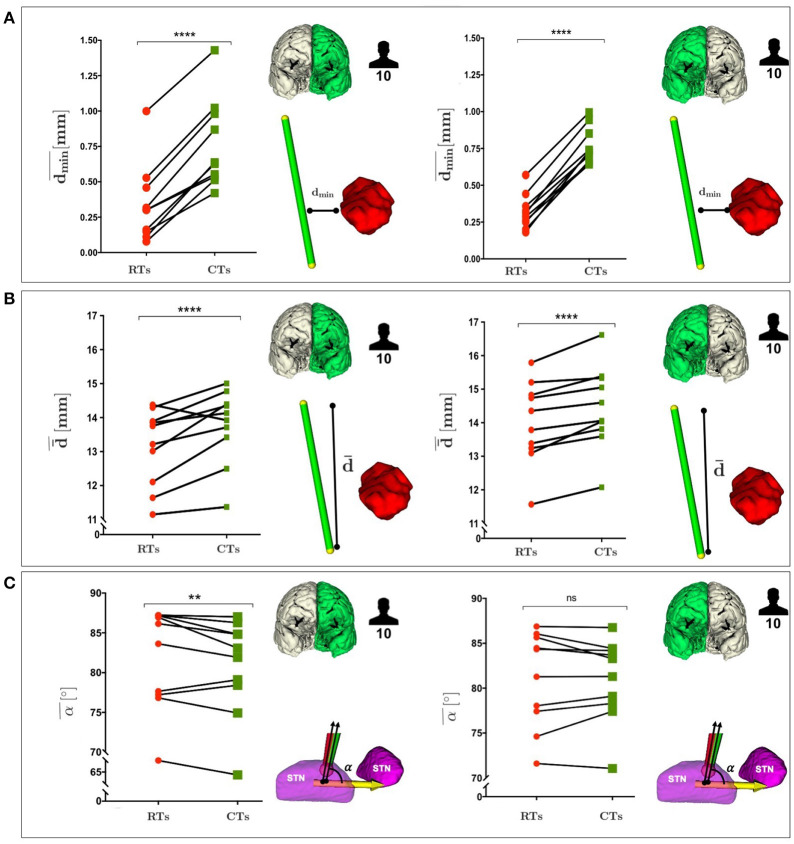
**(A)** Comparison between RTs and CTs, reported for the 10 subjects, in terms of the mean value of the *d*_*min*_, calculated over the best trajectory of all the EP_*i*_, from all critical AOs of left and right hemisphere. **(B)** Comparison between RTs and CTs, reported for the 10 subjects, in terms of the mean value of the $\bar{d}$, calculated over the best trajectory of all the EP_*i*_, from all critical AOs of left and right hemisphere. **(C)** Comparison between RTs and CTs, reported for the 10 subjects, in terms of the mean value of the STN entry angle, α, calculated over the best trajectory of all the EP_*i*_, from all critical AOs of left and right hemisphere. *P*-values were calculated using Wilcoxon matched-pairs signed rank test (**P ≤ 0.01, ****P ≤ 0.0001).

Figure 5 shows a comparison between RTs and CTs in terms of $\bar{d_{min}}$, $\bar{\bar{d}}$, and $\bar{\alpha }$, reporting for each subject the mean value of *d*_*min*_, $\bar{d}$, and α calculated over the best trajectory of all the EP_*i*_, from all critical AOs, of left and right hemisphere. As seen in Figure 5A and in Supplementary Figure S1A, CTs keep a significantly greater $\bar{d_{min}}$ from critical AOs with respect to RTs for all subjects in both the hemispheres (*P* ≤ 0.0001 left and right).

Remarkably, in Figure 5B and in Supplementary Figure S1B CTs also showed a statistically significant advantage over RTs as far as CTs were able to keep a greater $\bar{d}$ from critical AOs in both the hemispheres (*P* ≤ 0.0003 left and right).

In Figure 5C and in Supplementary Figure S1C, it could be observed the CTs minimization trend of α with respect to RTs in both the hemispheres.

**Figure 6:**
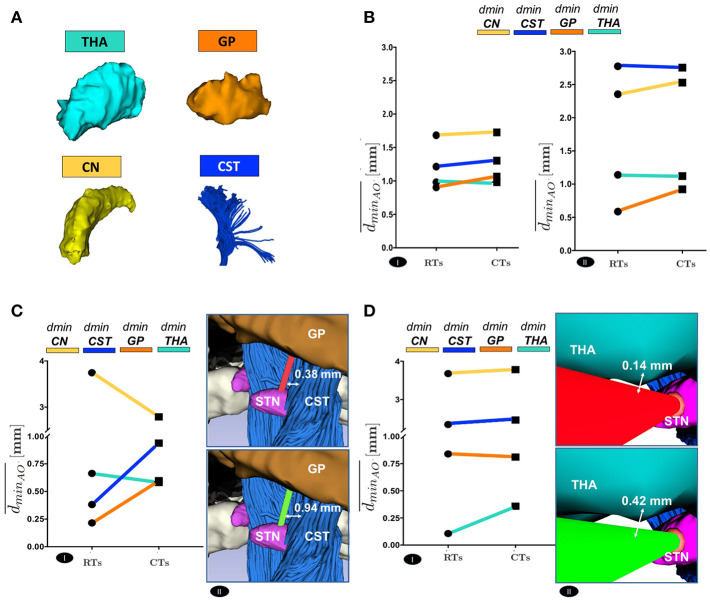
**(A)** The 3D Reconstruction of the most critical obstacles is shown. **(B)** Comparison between RTs and CTs, in terms of the mean value of the *d*_*mi*_*n*__*AO*__, calculated over the best trajectory of all the EP_*i*_ of all the subjects, from each AO separately, of left(I) and right (II) hemisphere. **(C)** Comparison between RTs and CTs, reported for 9647 subject left hemisphere, in terms of the mean value of *d*_*mi*_*n*__*AO*__, calculated over the best trajectory of all the EP_*i*_ of the subject, from each AO separately (I). The illustrative scene of 9647 single-case scenario has been taken from 3D Slicer 4.7.0 (II). **(D)** Comparison between RTs and CTs, reported for 5960 subject left hemisphere, in terms of the mean value of *d*_*mi*_*n*__*AO*__, calculated over the best trajectory of all the EP_*i*_ of the subject, from each AO separately (I). The illustrative scene of 5960 single-case scenario has been taken from 3D Slicer 4.7.0 (II).

The positive trend of maximized *d*_*min*_ can be globally appreciated even considering the delicate anatomical structures. Figure 6 shows a comparison between RTs and CTs in terms of $\bar{d_{min_{AO}}}$, reporting for each subject the mean value of *d*_*min*_ calculated over the best trajectory of all the EP_*i*_ from each AO ∈ THA ∨ GP ∨ CN ∨ CST of left and right hemisphere. As seen in Figure 6B and in Supplementary Figures S2A and S2B, if single structures are considered separately, only the minimal distance from GP optimized by CTs ($\bar{d_{min_{AO}}}$, *AO* ≡ *GP*) resulted statistically significant, while the improvement in the minimal distances from CN, CST and THA ($\bar{d_{min_{AO}}}$, *AO* ∈ {*CN* ∨ *CST* ∨ *THA*}, respectively) just followed a positive trend. This very likely depends on the fact that the algorithm optimizes every single case keeping a scenario-specific focus, balancing distances in different ways depending on the particular needs. Thus, averaging the distances from single structures of all the 10 subjects may flatten the effect of trajectory optimization. In fact, if single cases are considered, it is clear how every setting is unique and how the planner balances its computation accordingly. For instance, in subject #9647, RTs passed so critically near to GP and CST that the corresponding CTs should even reduce their distances to CN ($\bar{d_{min_{AO}}}$, *AO* ≡ *CN*) in order to maximize the minimal distance from GP and CST obstacles ($\bar{d_{min_{AO}}}$, *AO* ∈ {*GP* ∨ *CST*}, respectively) (*P* ≤ 0.01) (Figure 6C and Supplementary Figure S3A). Moreover, taking as another example subject #5960 in which THA is instead particularly threatened by RTs, it emerged how the algorithm could also ponder to move minimally closer to all the other structures in order to gain sufficiently safer minimal distance from the THA obstacle ($\bar{d_{min_{AO}}}$, *AO* ≡ *THA*) (*P* ≤ 0.01; Figure 6D and Supplementary Figure S3B).

Finally, we measured the electrode insertion angle with respect to the direction perpendicular to the skull surface. We recorded 99% success rate in inserting steerable electrodes in the skull with an angle < 30° (100% right, 98% left), while 98% success rate as far as the rigid electrodes were concerned (98% bilaterally).

**Figure 7:**
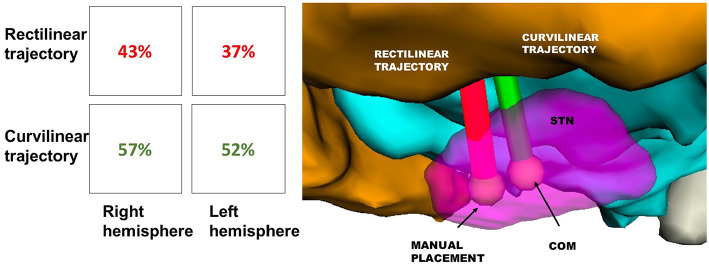
Success rate of RT(red) and CT(green) in reaching the STN, displayed in Table 1. The illustrative scene of single-case example has been taken from 3D Slicer 4.7.0 (II).

Moreover, after calculating all the possible trajectories, CTs reached the COM of STN with a success rate of 52% on the left and 57% on the right. On the other hand, feasible RTs that targeted the COM of STN just accounted for the 37% on the left and 43% on the right. Thus, between the tested trajectories, steerable electrodes could reach even this new TP more efficiently (Figure 7).

### 5.3. Computational Time

The computational time required to find the set of solutions {$\hat{\hat{curve^{i}}}$} for each EP_*i*_, *i* ∈ 1, .., 10 ranges from 1 to 3 min: such computational effort is required by the different steps of the workflow. All detailed data are reported in Table 3. Specifically, EOP is the most time consuming phase: the gradually smoothed path needs to repeatedly iterate in order to decrease its *d*_*min*_, $\bar{d}$ and *K*_*max*_, before reaching the final results.

**Table 3:** Results in term of computational time are shown.

**Computational time**			
**Step**	**25th**	**Median**	**75th**
RRT* [sec]	35.93	61.54	78.87
EOP [sec]	63.57	84.16	103.97
*F*_*cost*_ [sec]	2.02	2.05	2.45

## 6. Discussion

This work aims at developing a novel path planning approach for minimally invasive neurosurgery, in the context of EDEN2020. Although rectilinear DBS electrodes are now routinely exploited in the clinics (Deeb et al., 2016), the aim of our study is to demonstrate that the use of curvilinear electrodes can lead to the computing of safer trajectories that pass farther away from vulnerable anatomical obstacles. Some studies have recently demonstrated the advantages that potentially flexible alternatives could gain in terms of efficacy and safety, in the context of convention enhanced delivery of drugs (Engh et al., 2010), laser-driven amygdalohippocampectomy for epilepsy (Comber et al., 2017), and DBS for PD (Favaro et al., 2018b). The novelty of our planner consists in the possibility to consider as obstacles also white matter tracts depicted by advanced MR tractography, which is essential to avoid potential damages to pivotal functions. Fibers of the motor pathway have been considered in this specific setting due to the particularly hazardous position of the CST with respect to STN, the target of DBS for PD, but different white matter tracts could theoretically be integrated into a preoperative plan if other kinds of surgical procedures are performed, pointing to different TPs (Stypulkowski et al., 2017).

In this regard, future perspectives may include the exploitation of DBS in order to alleviate chronic pain such as peripheral neuropathic pain or cluster headache by directly stimulating the thalamus or the hypothalamus (Falowski, 2015). Given that PD does not alter the global brain architecture, especially in patients with preserved cognition for whom DBS is mostly useful (Seibyl et al., 2012), healthy volunteers have been selected for this computational study as a demonstration for the future inclusion of this advanced neuroimaging planning protocol for PD patients' evaluation. Indeed, since its timing is clinically compatible, dMRI acquisition for tractography reconstructions can be included in a preoperative DBS protocol. A possible concern might be the relatively small sample size of this study. However, the main aim of our work is to provide a proof-of-concept for the significant efficacy and clinical translatability of the proposed planner system, preliminary validating it in a restricted group of human subjects with the aim of expanding this cohort in future studies. Indeed, the concrete medical need that is addressed actually represents the main strength of our technical innovation in the field of artificial intelligence, as the practical advantages of our strategy emerge at the real interface between engineering and medical challenges. Furthermore, for the first time, state-of-the-art MRI methods including the newest diffusion MR Tractography technique have been integrated with an automatically computing trajectory planner that relies on sophisticated new steerable devices. The comprehensive MR imaging database exploited for the study is unique and distinctive, and will be publicly available at the end of the EU's Horizon EDEN2020 project. This database includes advanced diffusion MR imaging acquisitions for an enhanced dissection of white matter pathways in regions with higher microstructural brain tissue complexity, as well as high resolution morphological images providing exhaustive information on brain anatomy, arterial and venous vessels. Given the complexity of connecting all these features to the fully integrated system, the 10 real case-scenarios are necessary to support the technological innovation in this exploratory validation setting. The promising preliminary data support the feasibility of this approach and encourage its wide implementation in a larger cohort of patients to define its impact in a clinical setting.

### 6.1. Clinical Performance-Related Considerations

Different tests have been executed to evaluate the proposed path planning algorithm. The first study, performed over multiple EODs, demonstrates that solutions for curvilinear planning do exist even using an electrode larger than the usual ones. Limits imposed by RTs result less restrictive for CTs, opening up the possibility to consider different catheter designs for DBS (Amon and Alesch, 2017).

The second phase of investigations was performed on 10 subject, keeping the EOD and *K*_*max*_, respectively fixed to 1.3 *mm* and a *K*_*max*_ of 0.015 *mm*^−1^ and comparing CTs to RTs. The most important observation concerns the greater success in safeguarding pivotal anatomical obstacles by exploiting CTs instead of RTs. Curvilinear paths essentially find the best balance between all the structures, that must be considered altogether in their integer reciprocal complexity in order to fully appreciate the actual work of our algorithm. In fact, if single structures are analyzed unconnectedly, the focus on the calibrated equilibrium optimized for every single subject-specific anatomy may be lost. For instance, when a computed RT passes particularly near to one of the anatomical obstacles, the corresponding improved CT may even move closer to the other anatomical obstacles if this is necessary to ensure a minimal level of safety to the obstacle previously at extreme risk. Up to a limit of course, not to culminate in endangering another brain structure. Having elucidated this mechanism, it is clear how concentrating on a single anatomical obstacle may be misleading and how the advantages of CTs should be valued globally.

A further point is represented by the superior success rate reported by CTs in reaching the COM of the STN, that is hardly accessible by RTs. Overall, such notable results may be traced back to the combination of NURBS and GA implemented in CTs planning which demonstrates, on average, larger $\bar{d}$ and *d*_*min*_ (+145%, +22%) and an increased rate of success with respect to previous literature. As already quoted in the “Experimental setup” section, this study does not only concern the mathematical issue of automatically computing the COM of the nucleus, but it also encompasses relevant clinical implications. Human STN has been sub-parcellated in three functional sub-zones, of which the postero-mesial, including the COM, seems associated to pure motor functions (Accolla et al., 2014). Stimulating behind the classical anterior STN target is reported to offer statistically superior tremor benefit with respect to other targets (Ramirez-Zamora et al., 2016), probably due to the straight stimulation of at least one of the three identified hyperdirect pathways connecting the STN to Primary Motor Cortex (M1-motion execution), Supplementary Motor Area (SMA-motion planning), and Prefrontal Cortex (PFC-cognitive motor response selection) (Akram et al., 2017; Chen et al., 2018). In common clinical practice, since conventional MRI on 1.5T scanners hardly visualize the whole STN at a high resolution (Massey et al., 2012), it would be tough to precisely target its COM by manual planning. Conversely, taking advantages from the automatic planner and the possibility of computing CTs, this strategy could be concretely accomplished.

Moreover, another interesting aspect of our planner that can lead to clinically relevant advantages is the capability of minimizing the entry angle into the target, aiming to align the electrode with the main axis of STN. Even if statistically significant, it can be argued that a reduction of 1 or 2 degrees in the entry angle may not imply a huge gain in terms of stimulated STN area. Nonetheless, it should be taken into account that the STN is a very small structure (6 × 4 × 5 *mm* along the anteroposterior, mediolateral and dorsoventral axes, respectively; Richter et al., 2004), so even a minor improvement could be beneficial. Additionally, the strict curvature constraints that we considered refer to a particular electrode design but, if a different prototype with a greater flexibility is used, further optimization should be reached because the planner is implemented to look for it.

However, this tool may be useful when different surgical approaches are exploited in order to cure diverse pathologies, such as in occipital access for the amygdalohippocampectomy for epilepsy (Jermakowicz et al., 2017; Yin et al., 2017), or if the skull surface is bumpy or less easily accessible, such as in experimental approaches for reaching the hippocampus through the foramen ovale (Comber et al., 2017). Further validations are needed on real patients in order to understand if the aforementioned advantages can be gained even in actual clinical cases, but, globally, it can be stated that the new functions integrated in our algorithm allow the computing of extremely precise CTs for DBS, safer than ordinary RTs.

Eventually, speculating beyond the explored context of DBS, the remarkable benefits of the automated steerable path planning described in this work could potentially be exploited in many other clinical scenarios. First of all, computation of accurate curvilinear trajectories would allow the EDEN2020 programmable bevel-tip needle to reach deep inaccessible brain areas not only to stimulate targets or to feasibly ablate neuronal foci with aberrant activities, but also to deliver chemotherapy or targeted immunotherapy to brain tumors (Mamelak, 2005; Luther et al., 2014). In the second place, the technological impact of such an automated system could be reflected in the delivery of innovative local treatments for neurodegenerative disorders, such as β−amyloid degrading enzymes for Alzheimer's disease patients (Miners et al., 2011) or adenovirus-mediated gene therapy for Parkinson's disease (Sudhakar and Richardson, 2018). In conclusion, this automated steerable path planning system has a high impact potential on a variety of clinical applications, ensuring safety, and reproducibility to different microsurgical procedures.

### 6.2. Technical Evaluations

The proposed method represents a trade off between the pure optimality determined by methods such as graph-based approaches and the approximation obtained with sampling-based solutions. In the first case, the global optimality is reached at the cost of a computational time unbearable for a clinical scenario, even when real time responsiveness is not a requirement such as the case of a pre-operative neurosurgical planner. Our solution consists in the combination of a sampling-based approach with an EOP. The latter has the role of refining the computed path to obtain a quasi-optimal solution in a computational time consistent with the pre-operative surgical application for which the planner is designed. In fact, as stated by Razali and Geraghty (2011), although evolutionary optimization methods do not guarantee the global optimum, they can produce an excellent quasi-optimal solution without the high computational effort typical of graph-based approaches. To avoid the risk of falling into local minima when making a population of NURBS to evolve via the EOP, the Rank-based Roulette Wheel Selection method (Razali and Geraghty, 2011) is used for the selection of the parents to combine. This method has proved capable to reduce the risk for the algorithm to get trapped in local minima.

## 7. Conclusion

The present work proposes a novel automatic DBS planner developed as part of the EU's Horizon EDEN2020 project, with the goal of providing a state-of-the-art combined technology platform for minimally invasive surgery. The main innovation consists of integrating a new curvilinear trajectory approach for stereotactic implantation of DBS electrodes with cutting-edge neuroimaging planning, including advanced MR tractography to depict WM corticospinal tracts and semi-automatic medical image segmentation. Moreover, surgeons would have the possibility to express their individual preferences assigning different weights to the critical structures, creating a priority list for maintaining safe distances. Besides offering precious advantages also for standard RT computation, the great novelty of our work is the possibility to evaluate the safety and efficiency of steerable electrodes with respect to standard ones. CTs should be potentially able to overcome the limits imposed by the standard RTs in terms of minimum distance from critical gray and white matter obstacles. Accordingly, the possibility to perform CTs for STN targeting with the proposed algorithm gives us the opportunity to optimize all the fundamental aspects of the efficiency of the electrostimulation and, at the same time, to maximize the safeness of the therapy.

## Data Availability

The MRI datasets used for this study have been acquired in the framework of the European Union project EDEN2020 (https://www.eden2020.eu, grant agreement No. 688279) and will be made publicly available at the end of the project.

## Author Contributions

AS, VP, and AFav implemented the methods, performed the experiments, and drafted the manuscript. MR and AFal designed the clinical problem. ED and AC designed the concept idea and revised the manuscript.

### Conflict of Interest Statement

The authors declare that the research was conducted in the absence of any commercial or financial relationships that could be construed as a potential conflict of interest.

## Supplementary Material

Supplementary Figure S1 The population variability of all the distances reported in Figure 5 is reported in terms of interquartile range IQR=[25–75]. *P*-values were calculated using Wilcoxon matched-pairs signed rank test (***p* ≤ 0.01, *****p* ≤ 0.0001). **(A)** Comparison between RTs and CTs, reported for the 10 subjects, in terms of all the values of the *d*_*min*_ highlighting their population variability, calculated over the best trajectory of all the EPi, from all critical AOs of left and right hemisphere. **(B)** Comparison between RTs and CTs, reported for the 10 subjects, in terms of all the values of the $\bar{d}$ highlighting their population variability, calculated over the best trajectory of all the EP_*i*_, from all critical AOs of left and right hemisphere. **(C)** Comparison between RTs and CTs, reported for the 10 subjects, in terms of all the values of the STN entry angle, α, highlighting their population variability, calculated over the best trajectory of all the EP_*i*_, from all critical AOs of left and right hemisphere.

Supplementary Figure S2 The population variability of all the distances reported in Figure 6B is reported in terms of interquartile range IQR=[25–75]. *P*-values were calculated using Wilcoxon matched-pairs signed rank test (*****P* ≤ 0.0001). Comparison between RTs and CTs, in terms of all the values of the *d*_*mi*_*n*__*AO*__, calculated over the best trajectory of all the EP_*i*_ of all the subjects, from each AO separately, of left **(A)** and right **(B)** hemisphere.

Supplementary Figure S3 The variability between the 10 different selected trajectories starting from the 10 EPs of the single subject reported in Figures 6C,D is reported in terms of interquartile range IQR=[25–75]. *P*-values were calculated using Wilcoxon matched-pairs signed rank test (**P* ≤ 0.05). **(A)** Comparison between RTs and CTs, reported for 9647 subject left hemisphere, in terms of all the values of *d*_*mi*_*n*__*AO*__, calculated over the best trajectory of all the EPi of the subject, from each AO separately. The illustrative scene of 9647 single-case scenario has been taken from 3D Slicer 4.7.0. **(B)** Comparison between RTs and CTs, reported for 5960 subject left hemisphere, in terms of all the values of *d*_*mi*_*n*__*AO*__, calculated over the best trajectory of all the EPi of the subject, from each AO separately. The illustrative scene of 5960 single-case scenario has been taken from 3D Slicer 4.7.0.
