# Supplementary figures and images for: Automated Steerable Path Planning for Deep Brain Stimulation Safeguarding Fiber Tracts and Deep Gray Matter Nuclei
Source: Front Robot AI. 2019 Aug 6;6:70. doi: 10.3389/frobt.2019.00070 (PMC7806057; doi:10.3389/frobt.2019.00070)

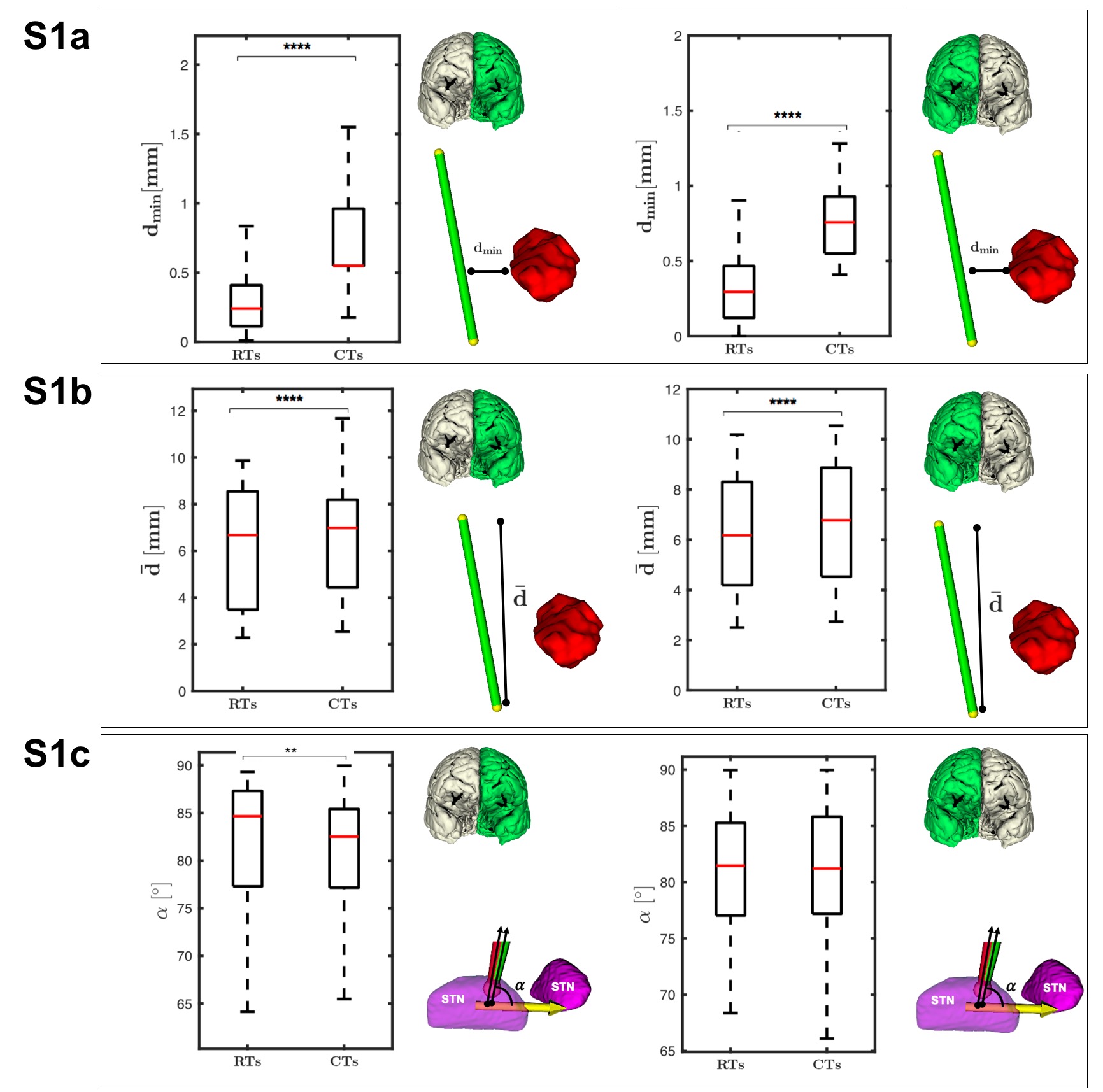

Supplement: Supplementary Figure S1 — The population variability of all the distances reported in Figure 5 is reported in terms of interquartile range IQR=[25–75]. P-values were calculated using Wilcoxon matched-pairs signed rank test (**p ≤ 0.01, ****p ≤ 0.0001). (A) Comparison between RTs and CTs, reported for the 10 subjects, in terms of all the values of the dmin highlighting their population variability, calculated over the best trajectory of all the EPi, from all critical AOs of left and right hemisphere. (B) Comparison between RTs and CTs, reported for the 10 subjects, in terms of all the values of the d¯ highlighting their population variability, calculated over the best trajectory of all the EPi, from all critical AOs of left and right hemisphere. (C) Comparison between RTs and CTs, reported for the 10 subjects, in terms of all the values of the STN entry angle, α, highlighting their population variability, calculated over the best trajectory of all the EPi, from all critical AOs of left and right hemisphere. [file Image_1.jpg]

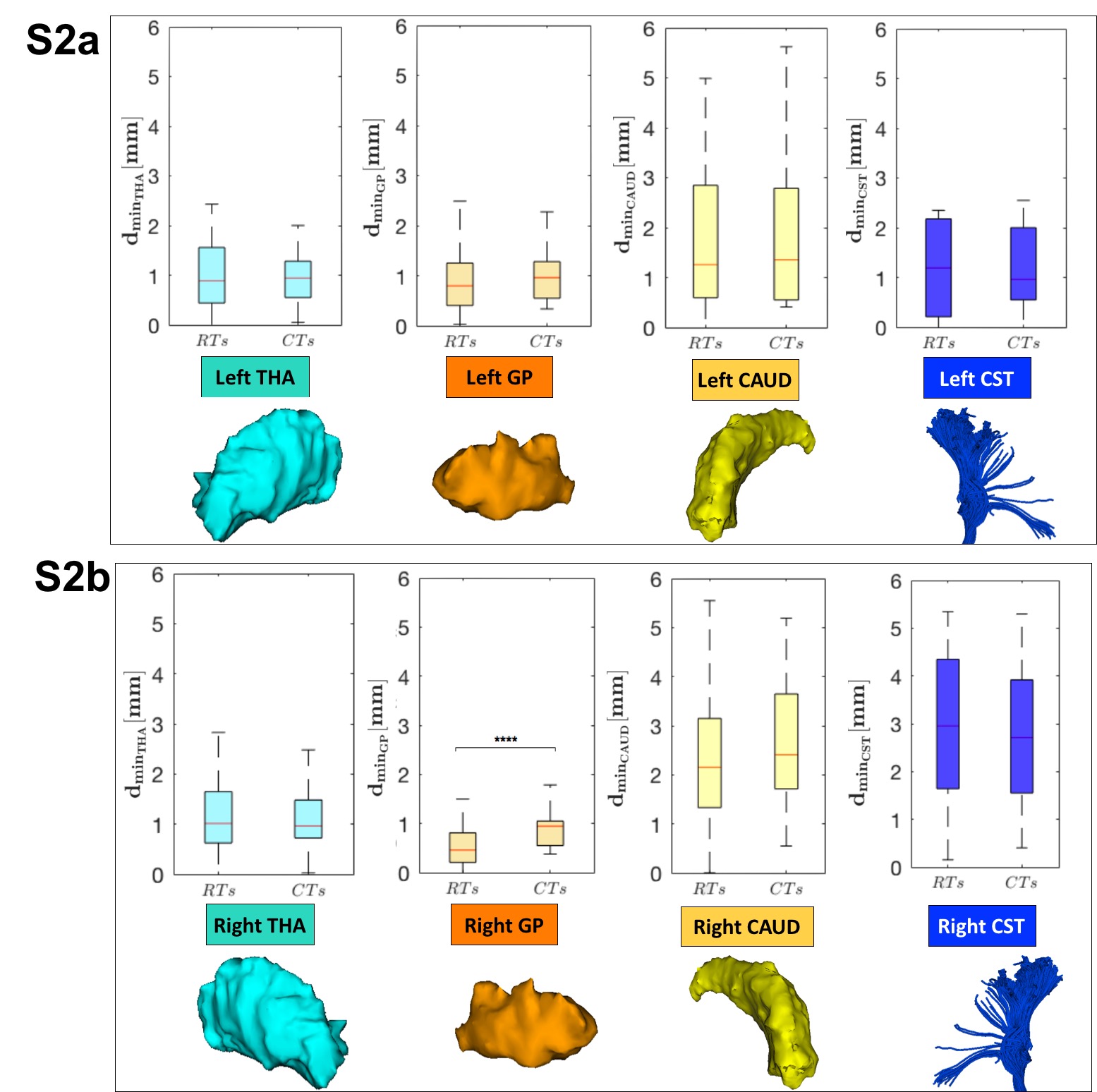

Supplement: Supplementary Figure S2 — The population variability of all the distances reported in Figure 6B is reported in terms of interquartile range IQR=[25–75]. P-values were calculated using Wilcoxon matched-pairs signed rank test (****P ≤ 0.0001). Comparison between RTs and CTs, in terms of all the values of the dminAO, calculated over the best trajectory of all the EPi of all the subjects, from each AO separately, of left (A) and right (B) hemisphere. [file Image_2.jpg]

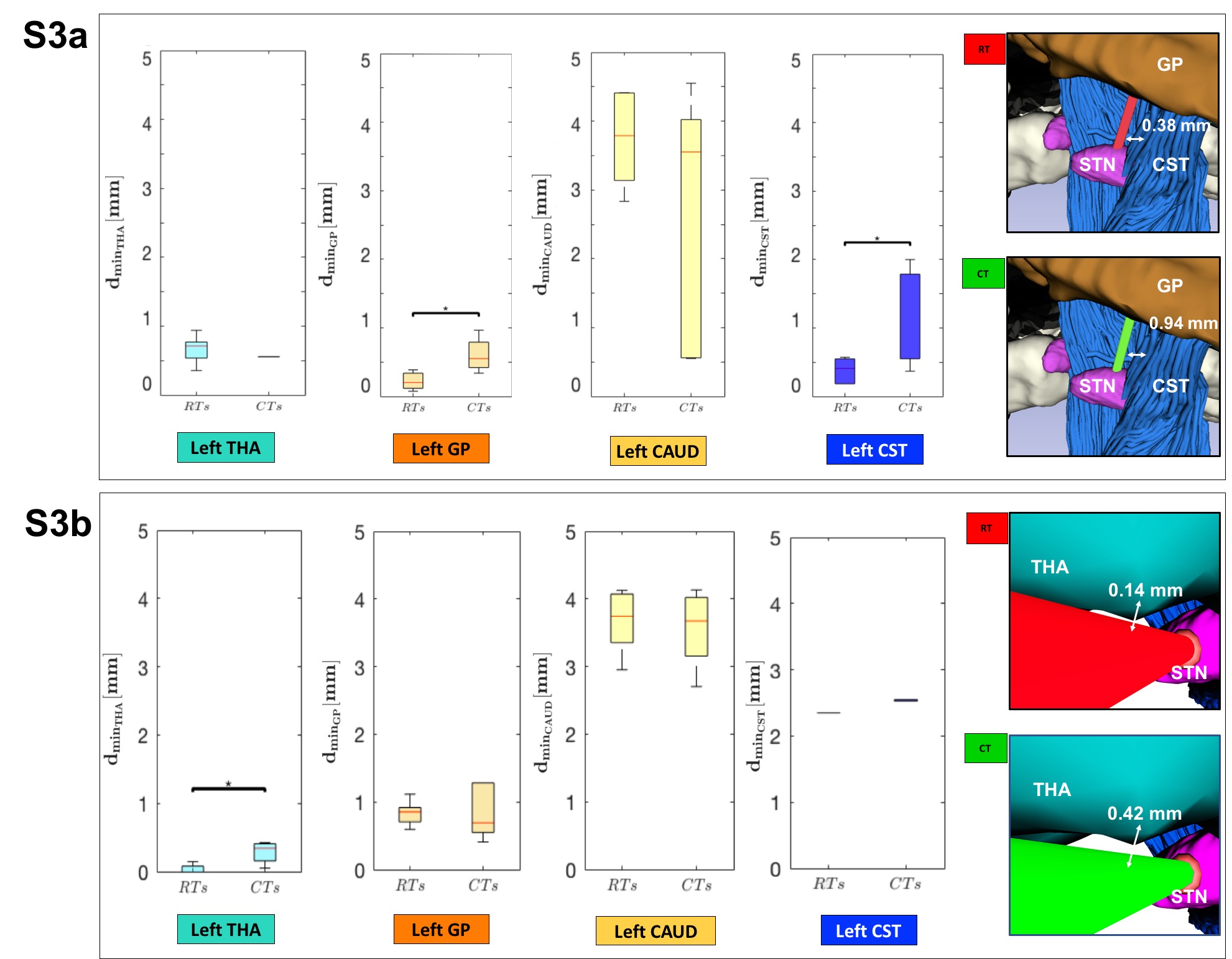

Supplement: Supplementary Figure S3 — The variability between the 10 different selected trajectories starting from the 10 EPs of the single subject reported in Figures 6C,D is reported in terms of interquartile range IQR=[25–75]. P-values were calculated using Wilcoxon matched-pairs signed rank test (*P ≤ 0.05). (A) Comparison between RTs and CTs, reported for 9647 subject left hemisphere, in terms of all the values of dminAO, calculated over the best trajectory of all the EPi of the subject, from each AO separately. The illustrative scene of 9647 single-case scenario has been taken from 3D Slicer 4.7.0. (B) Comparison between RTs and CTs, reported for 5960 subject left hemisphere, in terms of all the values of dminAO, calculated over the best trajectory of all the EPi of the subject, from each AO separately. The illustrative scene of 5960 single-case scenario has been taken from 3D Slicer 4.7.0. [file Image_3.jpg]
